# Genomic diversity, antimicrobial resistance and dissemination of *Serratia marcescens complex* in patients admitted to ICUs

**DOI:** 10.3389/fcimb.2025.1672468

**Published:** 2025-10-16

**Authors:** Wentao Zhu, Xi Chen, Hong Shen, Ming Wei, Chunxia Yang, Li Gu

**Affiliations:** Department of Infectious Diseases and Clinical Microbiology, Beijing Institute of Respiratory Medicine and Beijing Chao-Yang Hospital, Capital Medical University, Beijing, China

**Keywords:** *Serratia*, genomic surveillance, dissemination, antimicrobial resistance, ICU - intensive care unit

## Abstract

**Background:**

*Serratia* spp. is an important nosocomial pathogen, with increasing threat of antimicrobial resistance. We aimed to describe the population structure, antimicrobial resistance and dissemination of *Serratia* isolates in ICUs of China.

**Methods:**

*Serratia* spp. were isolated from patients admitted to ICUs of a large hospital between January 2014 and December 2024. Whole-genome and clinical data were collected to identify their epidemiological and evolutionary characteristics.

**Results:**

106 *Serratia* isolates was divided into five species based on phylogenomic and ANI analyses, namely *S. sarumanii*, *S. ureilytica*, *S. marcescens*, *S. bockelmannii*, and *S. nevei*. The predominant ST was ST595 (12.3%), followed by ST525 (10.4%) and ST428 (4.7%), all of which belonged to *S. sarumanii*. Based on a 16 SNPs threshold, 15 distinct clusters and 44 singleton strains were identified, with the largest cluster circulating in five different ICUs over the past 11 years. Notably, most grouped isolates within each cluster were isolated from different ICUs, indicative of potential inter-ICU transmission. The unique genes significantly enriched within each species contributed to their niche adaptation and plasticity. Various beta-lactamase genes, such as *bla*_CTX-M_ and *bla*_OXA,_ were detected, along with carbapenemase genes including *bla*_KPC-2_ and *bla*_NDM-5_ in nine isolates.

**Conclusion:**

These results contribute to understanding the population structure and dissemination of *Serratia* spp. in ICUs, highlighting their ongoing evolution towards increasing resistance and outbreak potential.

## Introduction

*Serratia* is a genus of Gram-negative, rod-shaped bacteria belonging to the family *Yersiniaceae* in the order Enterobacteriales (Adeolu et al., 2016). *Serratia* is considered to be ubiquitous in diverse environments, including water, soil, plants, and insects (Ono et al., 2022). However, it is not a common component of the human fecal microbiome (Bush and Vazquez-Pertejo, 2024). *Serratia* isolates can cause a range of nosocomial infections, including pneumonia, urinary tract infections, bacteremia, meningitis and endocarditis, particularly in immunocompromised patients (Mahlen, 2011). These infections account for about 1% to 2% of hospital-acquired infections (HAIs) and can lead to outbreaks with high morbidity and mortality rates, especially in pediatric or intensive care unit (ICU) wards (Mahlen, 2011; Aracil-Gisbert et al., 2024).

Taxonomically, the genus *Serratia* comprises twenty-four species (https://lpsn.dsmz.de/genus/serratia). *S. marcescens*, the most prevalent opportunistic human pathogen in this genus, frequently causes outbreaks of hospital-associated infections (Khanna et al., 2013; Van Goethem et al., 2024). Other members, such as *S. rubidaea* and *S. liquefaciens*, have also been reported to cause hospital-acquired infections, although less frequently (Dubouix et al., 2005; Karkey et al., 2018). Recently, a new species, *S. sarumanii*, was isolated from a wound swab (Klages et al., 2024). The transmission of some human-associated *Serratia* species via contaminated medical devices, such as nebulizers and catheters, is well known (Mahlen, 2011). However, most studies consist of individual case reports (Williams et al., 2022).

Clinical *Serratia* isolates are typically identified using MALDI-TOF MS (Aracil-Gisbert et al., 2024; Pérez-Viso et al., 2024; Van Goethem et al., 2024; Zhang et al., 2024). However, MALDI-TOF MS struggles to distinguish the species within *Serratia marcescens* complex (SMC), including *S. marcescens*, *S. ureilytica*, *S. bockelmannii* and *S. nematodiphila* (Harch et al., 2025). Furthermore, newly proposed species, such as *S. montpellierensis* (Blackburn et al., 2024) and *S. sarumanii* (Klages et al., 2024), may be sufficiently distinct at the molecular level, yet are not represented in current MALDI-TOF MS databases. Therefore, MALDI-TOF MS cannot reliably differentiate species within the SMC and should be complemented by whole genome sequencing (WGS) for species identification (Harch et al., 2025).

In this work, we aimed to provide a comprehensive understanding of *Serratia marcescens* complex in the ICUs of our hospital over an 11-year period (2014–2024). Based on WGS, we conducted a large-scale precision epidemiology analysis to decipher the evolution of clinical *Serratia marcescens* complex. Additionally, we compared the genomic features, in-hospital transmission patterns, antimicrobial resistance, and clinical characteristics of *Serratia marcescens* complex across different species.

## Materials and methods

### Study design and bacterial isolates

This retrospective study investigated inpatients infected with *Serratia marcescens* complex at Beijing Chao-Yang Hospital of Capital Medical University, a 2,500-bed public teaching hospital located in the capital of China that receives approximately four million patients per year. The study enrolled *Serratia marcescens* complex isolated from inpatients admitted to the ICUs from January 2014 to December 2024. Firstly, samples from patients suspected of having a bacterial infection were collected and send to the clinical microbiology laboratory for pathogen isolation and identification. The clinical samples included sputum, bronchoalveolar lavage fluid (BLAF), urine, drainage, body fluid, and wound. Suspected isolates that were cultured on China blue agar plates (Product code: HBPM6233, Thermo, USA) were initially identified using MALDI-TOF MS. Secondly, initial inclusion criteria varied across sample types. For non-sterile samples, such as sputum and BLAF, inclusion was only considered when the number of *Serratia marcescens* complex in the sample accounted for the vast majority. For sterile samples, inclusion was only considered when the number of *Serratia marcescens* complex in the sample was higher than 10^5^ CFU/mL. Thirdly, the final inclusion criteria required that the patients received the appropriate treatment for the *Serratia marcescens* complex infection, as determined based on their electronic medical records. In total, non-duplicate isolates were mainly recovered from sputum (n = 89), followed by BLAF (n = 6), urine (n = 5), ascites (n = 3), drainage (n = 2), and wound (n = 1).

### Antimicrobial susceptibility testing

Antimicrobial susceptibility testing of the bacterial isolates was investigated using the VITEK 2 system (bioMérieux, France) with GN09 cards following the guidelines provided by the Clinical and Laboratory Standards Institute (CLSI, 2025). In addition, the susceptibility of several antimicrobial agents, such as meropenem and imipenem, were determined or confirmed using the Kirby-Bauer method. *Escherichia coli* ATCC 8739 was used as the reference strain for quality control in all procedures. A total of 17 antimicrobial agents were tested, including amikacin (AK), aztreonam (ATM), ceftazidime (CAZ), Ciprofloxacin (CIP), gentamicin (CN), ceftriaxone (CRO), cefepime (FEP), imipenem (IPM), levofloxacin (LEV), meropenem (MEM), minocycline (MH), piperacillin (PRL), cefperazone-sulbactam (SCF), sulfamethoxazole-trimethoprim (SXT), tigecycline (TGC), tobramycin (TOB), and piperacillin-tazobactam (TZP).

### Whole-genome sequencing and annotation

The genomic DNA of all bacterial isolates was extracted using Wizard Genomic DNA extraction kits (Promega, Wisconsin, USA) and stored at −80 °C until shipment to the Shanghai Majorbio Bio-pharm Technology Co., Ltd for whole genome sequencing on an Illumina HiSeq 2500 platform, resulting in a 2×150 bp paired-end library for each isolate. The quality control of raw sequenced reads was conducted as previously reported (Liu et al., 2025), which was used to eliminate sequences with low-quality (Q ≤ 20) and adapters. The clean data obtained was *de novo* assembled to generate draft contigs using SPAdes v4.1.0 (Prjibelski et al., 2020). The gaps of draft contigs were further filled using gapclose v1.12, resulting in draft genomes (Xu et al., 2020). Whole genome annotation, such as potential open reading frames and their functions, was investigated using Prokka pipeline v1.14.5 (Seemann, 2014). Taxonomic classification was determined using the GTDB Toolkit, which was based on the Genome Database Taxonomy (GTDB) (https://gtdb.ecogenomic.org/). The reference genomes for *S. marcescens*, *S. ureilytica*, *S. nevei*, *S. bockelmannii*, and *S. sarumanii* in the GTDB were GCF_017299535.1, GCF_013375155.1, GCF_008364245.1, GCF_008011855.1, and GCF_001537005.1, respectively. The species of sequenced genome was also identified using the rMLST scheme as previously proposed (Aracil-Gisbert et al., 2024; Harch et al., 2025), which is available at https://pubmlst.org/species-id.

### Comparative genomic analysis

The sequence type (ST) of genomes was determined using PubMLST 7-loci schema (https://pubmlst.org/organisms/serratia-spp). Unassigned allele sequences were extracted and submitted to BIGSdb (Jolley and Maiden, 2010), and obtained 47 new STs. The whole-genome average nucleotide identity (ANI) was calculated as previously described (Bonnin et al., 2025), with intra-species strains displaying > 95% ANI. Antimicrobial resistance genes were identified using abricate v1.0.0 based on the NCBI AMRFinderPlus database (Feldgarden et al., 2019). The intrinsic genes were determined according to a previous report (Sandner-Miranda et al., 2018). The identified antimicrobial resistance genes were further classified into drug class based on the Comprehensive Antibiotic Resistance Database (CARD) (Alcock et al., 2020). Virulence genes were screened using abricate v1.0.0 based on virulence factor database (VFDB) with 80% coverage and 80% identity (Chen et al., 2016; Liu and Zhu, 2025; Zhu et al., 2025). The GFF3 format of each genome was obtained from Prokka annoation result and used to conduct core-pan genome analysis using the Roary pipeline v3.12.0 (Page et al., 2015). The *Serratia* core genome was defined by sequences present in ≥ 95% of these genomes as previously reported (Näpflin et al., 2025; Zhu et al., 2025).

### Phylogenetic analysis

Single nucleotide polymorphism (SNP) analysis was conducted using Snippy v4.6.0 with default parameters (https://github.com/tseemann/snippy), utilizing the first identified genome (CY27690) from this study as the reference. The core genome alignment was then refined to eliminate elevated densities of base substitutions and recombination events using Gubbins v3.4 (Croucher et al., 2015). The core genome SNP (cgSNP) distance matrix of all paired genomes was calculated using snp-dists (https://github.com/tseemann/snp-dists). Paired genomes differing by no more than 16 SNPs were categorized as possible outbreaks (cluster groups) in the hospital, according to the previously recommended threshold (Chng et al., 2020), and were visualized using Standard Neighbour Joining algorithm of GrapeTree of (https://github.com/achtman-lab/GrapeTree). The maximum likelihood tree, based on core genome alignment, was built using IQtree v2.0.6 (https://github.com/Cibiv/IQ-TREE) and visualized using the Interactive Tree of Life (iTOL) web server (https://itol.embl.de/).

### Statistical analysis

The clinical symptoms and demographic data were retrospectively extracted from the electronic medical record system. Categorical variables, such as age groups, were compared using the median and interquartile range (IQR). Violin plots were obtained using ggplot2 package. *P* values of < 0.05 indicated statistical significance. All statistics analyses were performed using R software package v4.3.3 (https://www.r-project.org/).

## Results

### Spatiotemporal distribution of *Serratia marcescens* complex in the ICUs

From January 2014 to December 2024, a total of 874 *Serratia* strains were isolated from all departments. The prevalence of *S. marcescens* complex in all bacterial strains isolated from the whole hospital ranged from 0.40% to 3.54% per year, with the highest prevalence occurring in 2014 (**Supplementary Figure S1A**). Among all the *Serratia* strains, the prevalence of these *S. marcescens* complex isolated from the ICUs was 4.6% to 30.4% per year, with the highest prevalence seen in 2024 (**Supplementary Figure S1B**). This suggested that the prevalence trend of *S. marcescens* complex in all bacterial strains was the opposite of that of *S. marcescens* complex from the ICU in *Serratia* strains per year (**Supplementary Figure S1**). In total, 106 non-repetitive *Serratia* spp. were included for further analysis (**Supplementary Data Sheet 0**), with isolation coming from five different ICUs: cardiac ICU (CICU, n = 8), emergency ICU (EICU, n = 18), neurology ICU (NICU, n = 30), respiratory ICU (RICU, n = 23), surgical ICU (SICU, n = 27).

### Population structure of *Serratia marcescens* complex in the ICU

**Figure 1:**
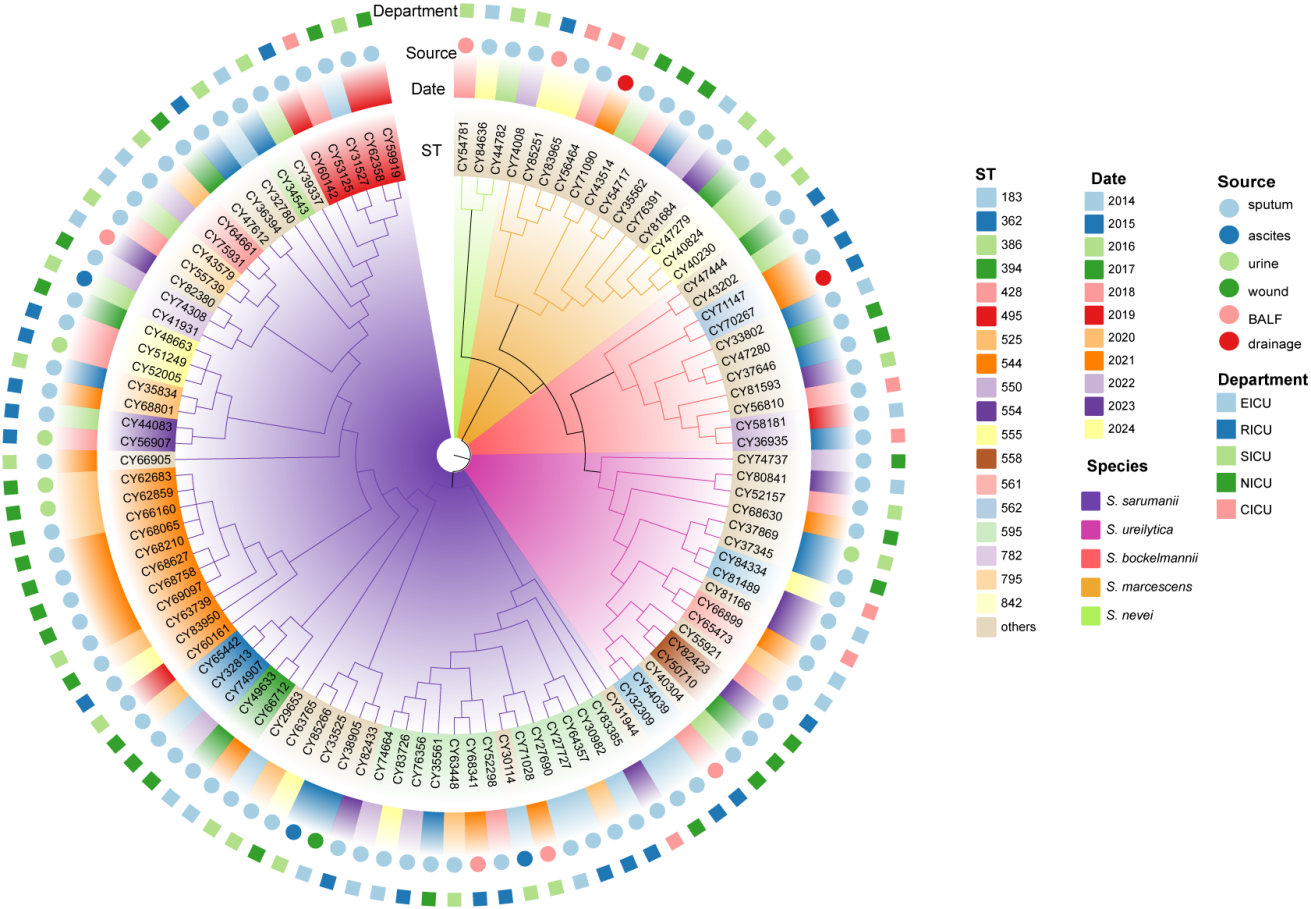
Phylogenetic analysis of *Serratia* clinical isolates based on core genome. The maximum-likelihood phylogenetic tree was constructed using 3151 core genes that were presented in ≥ 99% of genomes. The branch colors are labeled with corresponding species. The colored rings, from the inside out, represent the ST, collection date, source, and department of the strains isolated, respectively. BALF, bronchoalveolar lavage fluid; CICU, cardiac ICU; EICU, emergency ICU; NICU, neurology ICU; RICU, respiratory ICU; SICU, surgical ICU.

All the 106 *Serratia* spp. were initially identified as *S. marcescens* by the standard method in clinical labs (MALDI-TOF MS). To further confirm their species classification and genetic diversity, the whole genomes of these isolates were sequenced. The core genome among these isolates revealed a total of 469,717 core SNPs that were shared. Phylogenomic analysis based on core genome alignment indicated that the 106 *Serratia* isolates were clustered into five groups (**Figure 1**). ANI analysis of these 106 *Serratia* isolates also showed a diverse population structure, forming five groups (**Supplementary Figure S2**). The ANI values between different groups were all below the generally accepted 95-96% ANI threshold, suggesting that the five groups belonged to five different species, including *S. sarumanii* (n = 62), *S. ureilytica* (n = 17), *S. marcescens* (n = 13), *S. bockelmannii* (n = 11), and *S. nevei* (n = 3).

The *in silico* MLST analysis was performed utilizing the established *Serratia* spp. scheme. In total, these *Serratia* strains were categorized into 62 STs, with 43 STs represented by only one isolate each. The most prevalent ST was ST595 (12.3%, 13/106), followed by ST525 (10.4%, 11/106), ST428 (4.7%, 5/106), ST362 (2.8%, 3/106), ST554 (2.8%, 3/106), and ST842 (2.8%, 3/106). The ST diversity of *Serratia* strains from inpatients admitted to the SICU was more pronounced (**Supplementary Figure S3**). Notably, over a quarter of the isolates from patients in the NICU and RICU belonged to ST525 (26.7%) and ST595 (26.1%), respectively. Furthermore, all ST525 and ST595 isolates belonged to *S. sarumanii* (**Figure 1**). Interestingly, the STs of all the isolates from the CICU were different.

### Genomic diversity of *Serratia marcescens* complex in the ICUs

**Figure 2:**
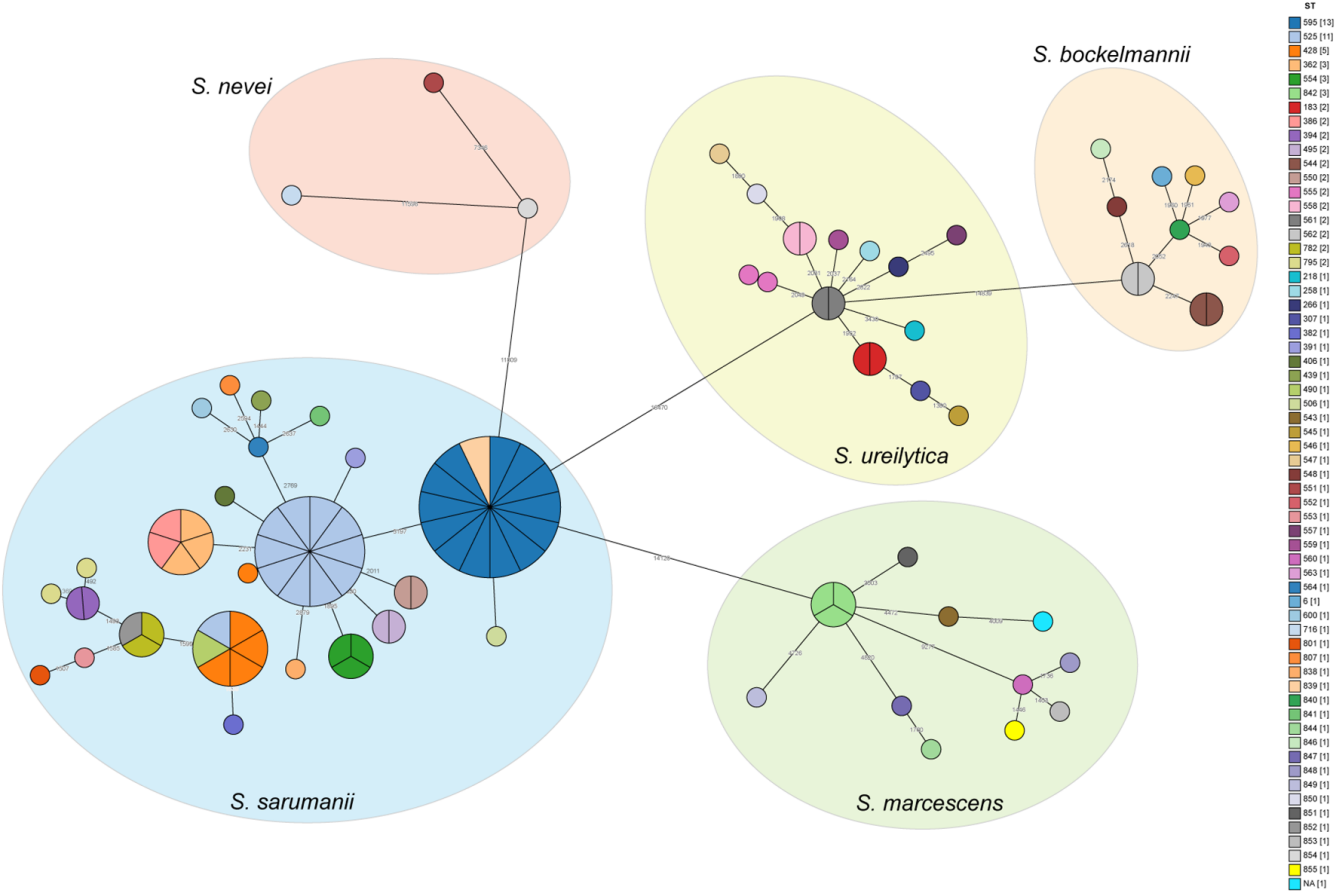
Minimum spanning trees showing the SNP difference among *Serratia* clinical isolates. Strains are grouped together based on having less than 16 SNPs. Each circle or section of the pie chart represents an individual strain, with the color indicating its ST. The number of SNPs between strains are showed on the connecting lines. The numbers in brackets in the legend indicate the number of strains in each ST.

**Figure 3:**
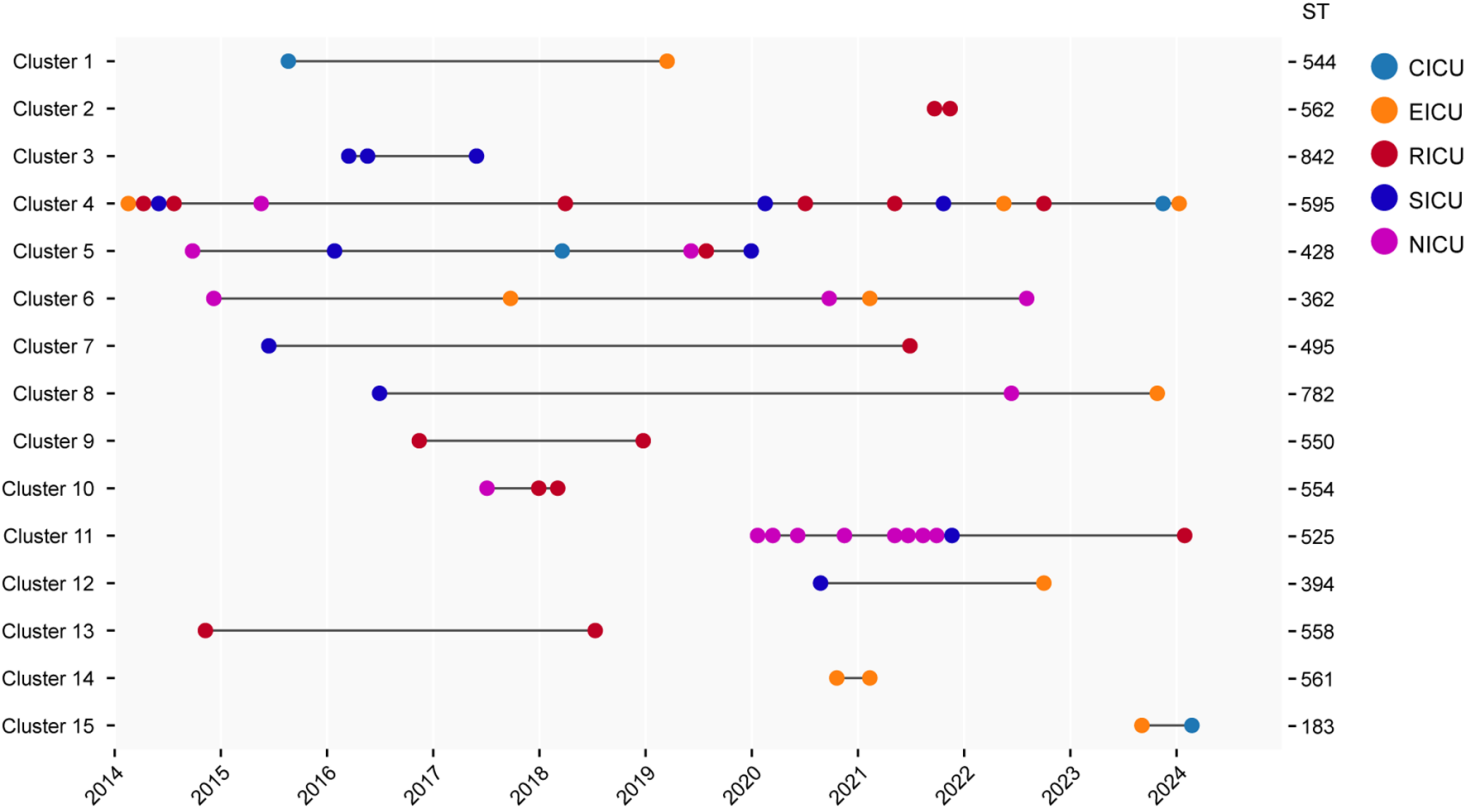
Timeline of detected cases in each cluster. The left Y axis is labeled with the name of each cluster, and the right Y axis is labeled with the ST of corresponding cluster. The X axis is labeled with collection year. CICU, cardiac ICU; EICU, emergency ICU; NICU, neurology ICU; RICU, respiratory ICU; SICU, surgical ICU.

The pairwise SNP distance matrix was estimated based on the core genome mentioned above, and used to construct a minimum spanning tree (MST). The isolates belonged to separate lineages that were identical to the species classification (**Figure 2**). The number of pairwise SNPs among the analyzed *Serratia* isolates ranged from 0 to 17723, with all isolates sharing a median of 3300 (IQR 3,197-14,535) SNPs with the first isolated strain (CY27690). Based on the threshold of 16 SNPs, these 106 isolates were assigned to 15 distinct genetic clusters (GCs) and 44 singleton strains. The majority of GCs were comprised of only two isolates (IQR 2-3). The largest GC was cluster 4, which included thirteen ST595 isolates and one ST839 (new ST identified in this study) isolate (CY83385) (**Figure 3**). These strains from cluster 4 circulated in five different ICUs over the past 11 years (**Figure 3**). Cluster 11, the second largest transmission chain, contained ten isolates from ST525, which were mainly prevalent in NICU between 2020 and 2021 before spreading to SICU in 2021 and RICU in 2024. Cluster 5 contained six isolates from three different STs (ST428, ST490, and ST525), spreading among four ICUs between 2014 and 2020. Cluster 6 had only five isolates from two ICUs, which belonged to two STs, including two from ST368 and three from ST362. Of particular interest was that isolates in each cluster were collected over a span of at least two different years (**Supplementary Figure S4**), suggesting the duration for which the strains had been circulating. Overall, most clustered isolates in each cluster were isolated from different ICUs, indicating of potential outbreaks and transmission.

### Analysis of accessory genome

**Figure 4:**
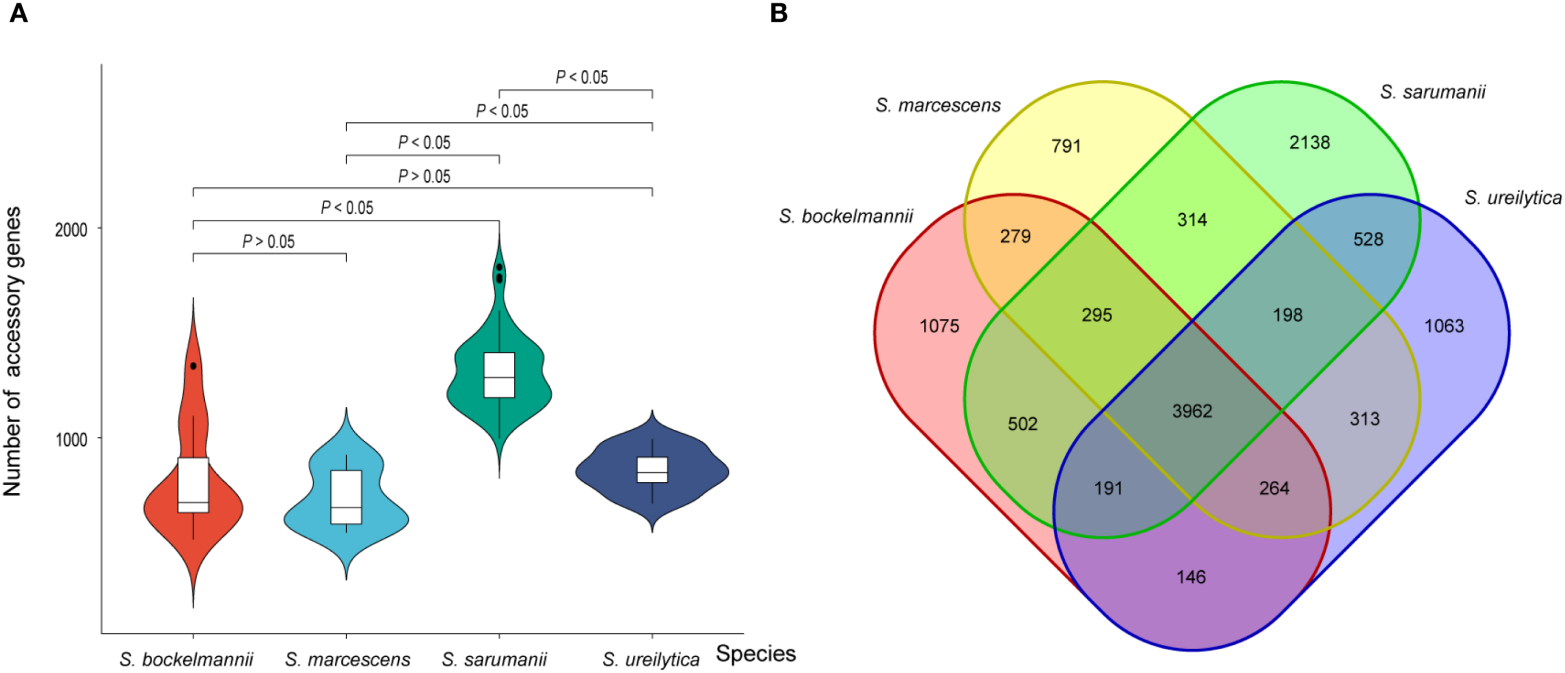
**(A)** Violin plot displaying the number of accessory genes for each species **(A)**. The horizontal line within each box plot represents the median number of accessory genes for each species. Significance values are labeled on the top of plot. **(B)** Venn plot illustrating the shared accessory genes among each species.

Accessory genes were analyzed to determine the presence of adaptive traits across different species. As the number of genomes examined increased, the number of core genes remained relatively stable, while the number of accessory genes showed an increasing trend (**Supplementary Figure S5**). Among 14242 (80.6%) accessory genes identified, less than 95% were shared across all *Serratia* spp. The average number of accessory genes per isolate within each species was determined (**Figure 4A**; **Supplementary Data Sheets 2–5**), with the highest number found in isolates of *S. sarumanii* (*P* < 0.05). Unique genes were notably enriched in isolates of *S. sarumanii* (n = 1075), the most represented species in the study, followed by *S. ureilytica* (n = 1063) (**Figure 4B**). Of particular interest within all *S. marcescens* isolates were the enrichment of *adh3*, coding for alcohol dehydrogenase, and *fosB2*, which imparts resistance to the antibiotic Fosfomycin. Moreover, isolates of *S. sarumanii* were enriched in various proteins, including hemolysin transporter protein ShlB, macrolide export ATP-binding/permease protein MacB, toxic protein HokC that required for toxin and drug export, as well as proteins associated with type II secretion system like type II secretion system protein E J, and L.

### Antimicrobial susceptibility and resistance phenotypes

**Figure 5:**
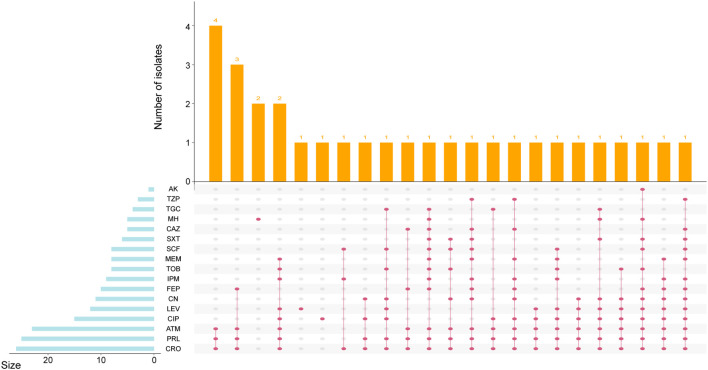
UpSetR plot showing the resistance phenotype of *Serratia* clinical isolates. The left histogram indicates the number of isolates with each antimicrobial resistance. The top histogram reveals the distribution of isolates by type. The phenotypic results indicate resistance as defined by the Clinical and Laboratory Standards Institute guidelines.

The antimicrobial susceptible testing revealed that a total of 76 (71.7%) isolates were susceptibility to all 17 antimicrobial agents tested (**Supplementary Data Sheet 1**). The resistance of the remaining 30 strains ranged from 1 and 13 antimicrobial agents per isolate, with a median of 5 (IQR 3-9) (**Figure 5**). Among these *Serratia* spp., the most common resistance was observed against CRO (24.5%, 26/106), followed by PRL (23.6%, 25/106), ATM (21.7%, 23/106), CIP (14.1%, 15/106), LEV (11.3%, 12/106), CN (10.4%, 11/106), and FEP (9.4%, 10/106). Notably, nine isolates were resistant to IPM (8.5%, 9/106), indicating the emergence of carbapenem-resistant *S. marcescens* complex. The prevalence of other resistance phenotypes was all less than 10% (< 9 isolates). The most prevalent resistant profile included resistance to CRO, PRL, and ATM simultaneously. The resistant profile of most antimicrobial resistant isolates was unique among *Serratia* spp. in this study. Furthermore, isolates demonstrating antimicrobial resistance to the tested agents were primarily presented in *S. sarumanii* (83.3%, 25/30) and *S. bockelmannii* (10.0%, 3/30).

### Patterns of antimicrobial resistance and virulence genes

**Figure 6:**
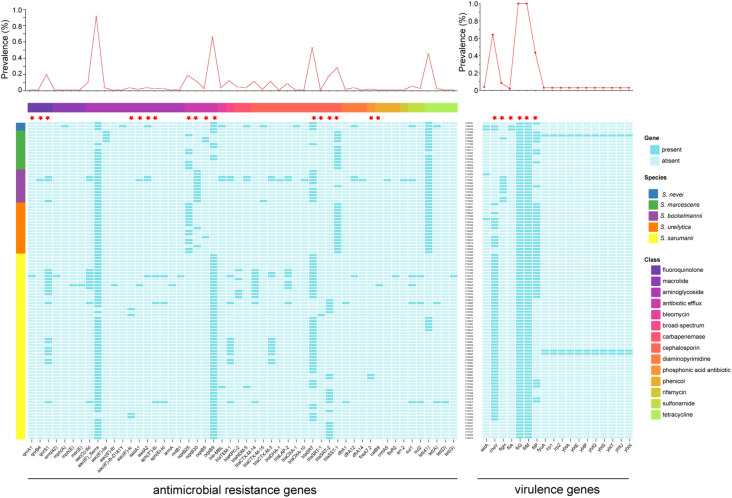
Distributions of antimicrobial resistance genes and virulence genes. The line chart at the top showed the incidence of each gene. The color of left rectangle represents the corresponding species, while the color of top rectangle is labeled with the corresponding drug class of antimicrobial resistance genes. The intrinsic antimicrobial resistance and virulence genes were labeled with red five-pointed stars.

To investigate their resistome, genomes were screened for the presence of antimicrobial resistance (AMR) genes (**Figure 6**). A total of 52 AMR genes were identified from the 106 *Serratia* isolates, which were classified into 14 drug classes. The number of AMR genes varied across isolates, ranging from 3 to 21, with a median of 4 (IQR 3-4). The most prevalent AMR gene was intrinsic *aac(6’)_Serra* gene (97/106), which was prevalent in all five species. However, the second most prevalent AMR gene, intrinsic *oqxB9* gene (70/106), was absent in isolates belonging to *S. bockelmannii* and *S. ureilytica*, and present in several isolates of *S. marcescens*. The strains carrying AMR genes associated with cephalosporin, aminoglycoside, and antibiotic efflux were distributed in all isolates. Among 12 beta-lactamase genes conferring resistance to cephalosporin, intrinsic *bla*_SRT_ gene was the most prevalent (56/106). Additionally, three acquired *bla*_CTX-M_ genes, including *bla*_CTX-M-14_ (n = 12), *bla*_CTX-M-3_ (n = 12), and *bla*_CTX-M-15_ (n = 2), were prevalent in 26 isolates, which was consistent with the results of the CRO resistance genotypes. In addition, acquired carbapenemase genes were detected in nine *Serratia* isolates (8.5%, 9/106), including five *bla*_KPC-2_ (4.7%, 5/106) and four *bla*_NDM-5_ (3.8%, 4/106), which aligned with the results of the IPM resistance phenotypes.

The potential pathogenicity of *Serratia* spp. in the ICUs were determined based on the distribution of virulence genes (**Figure 6**). The most common virulence-related genes were intrinsic *fliG* and *fliM* genes, which were prevalent in all isolates. And, the intrinsic *cheY* gene, involved in the transmission of sensory signals to the flagellar motors, was the second common virulence-related gene (64.2%, 68/106), followed by intrinsic *fliP* gene (43.4%, 46/106), which were sporadically distributed across species. It is noteworthy that three isolates contained a high-pathogenicity island comprised of *fyuA*, *irp1*, *irp2*, *ybtA*, *ybtE*, *ybtP*, *ybtQ*, *ybtS*, *ybtT*, *ybtU*, *ybtX*. Furthermore, there was a significant difference in the number of virulence genes between several species (**Supplementary Figure S6**), as well as between isolates of several clusters (**Supplementary Figure S7**).

### Clinical manifestations of *Serratia marcescens* complex in the ICU

To evaluate the differences in clinical manifestations, we retrieved the electronic medical records of inpatients infected with *Serratia* spp. The median age of all cases was 69 years (IQR 60-79), with 68.9% of them being male (73/106). We proceeded to compare the clinical manifestations caused by the four main species (**Supplementary Table S1**). Although the proportion of male patients infected with *S. ureilytica* seems to be similar to that of female patients, a larger proportion of males were infected with the remaining three species, with a significant difference noted in *S. sarumanii* (*P* = 0.0001). The median age of patients diagnosed with *Serratia* infection showed a significant difference among four species (*P* < 0.05), with the median age of patients diagnosed with *S. bockelmannii* being the youngest. Individuals infected with *S. sarumanii*, *S. ureilytica* and *S. marcescens* tended to be older. The clinical symptoms including fever (median 38°C, IQR 37.6-38.8°C) and cough had a higher prevalence. The incidence of severe pneumonia ranged from 16.7-28.6%. Dyspnea and chill were only observed in patients infected with *S. sarumanii* and *S. ureilytica*. The abnormality rate of clinical examination varied, with the highest WBC count observed in cases of *S. marcescens* and *S. bockelmannii*, neutrophilic granulocyte percentage in *S. marcescens*, and hemoglobin in *S. sarumanii*.

## Discussion

Although a wide variety of species have been proposed in the genus *Serratia*, most of the understanding of *Serratia* has focused on the type species, *S. marcescens* (Williams et al., 2022). This study retrospectively investigated the *Serratia* species isolated from multiple sources, revealing the wide genomic diversity of human-associated *Serratia* within five ICU wards over the past 11 years. These *Serratia* spp. were genomically classified into five species, with the majority of them being *S. sarumanii*, which was identified as a novel species in the genus *Serratia* in 2024 (Klages et al., 2024). The findings of this study indicated that there were multiple species of *Serratia* can infected humans, and similar species may not be accurately distinguished by MALDI-TOF MS, but could be differentiated by conserved genomic regions.

Analysis of closely clustered isolates using cgSNP distance matrix with a threshold of 16 SNPs (David et al., 2019) revealed a high incidence of clonal dissemination of *Serratia* spp. Our study also showed that the largest cluster had spread over 11 years, including inter-ICU transmission, which exceeded previous reports (Zhang et al., 2024). Multiple simultaneous transmission chains were identified within the same ICU, raising concerns about ongoing dissemination. However, the full extent of *Serratia* spp. dissemination may be underestimated as not all isolates collected from 2014 to 2025 were included, nor those obtained prior to 2014. The exact routes of transmission remain unclear, but potential sources could include contact with caregivers, cleaning workers, shared bathrooms, and changes in hospital departments during a patient’s stay. In addition, more than 11,500 SNPs were identified between one isolate and the other two isolates within *S. nevei*, indicating the potential existence of a sixth group.

Both intrinsic and acquired AMR genes contributed to antibiotic resistance across *Serratia* species. The chromosomal AmpC-type *β*-lactamase genes, including *bla*_SRT_, *bla*_SRT-1_,and *bla*_SRT-2_, were present in all isolates, with each isolate having a resistance gene, consistent with previous research (Matteoli et al., 2021). The acquired *β*-lactamase genes in this study, such as *bla*_CTX-M_ and *bla*_OXA_, were found primarily in *S. sarumanii* species. Notably, carbapenemase-producing *Serratia* spp. were detected, with *bla*_KPC-2_ found exclusively in *S. sarumanii*. Additionally, the carbapenemase gene, *bla*_NDM-5_, was prevalent in three *Serratia* species. Although previous reports have described the presence of *bla*_KPC-2_, *bla*_VIM-1_, and *bla*_OXA-48_ in *Serratia* spp (Matteoli et al., 2021; Pérez-Viso et al., 2024; Tang et al., 2024; Zhang et al., 2024), NDM-5-producing *Serratia* isolates have been rarely reported in China or elsewhere (Matteoli et al., 2021; Nakanishi et al., 2022; Pérez-Viso et al., 2024). Fortunately, none of these carbapenemase-producing *S. marcescens* complex caused clonal outbreaks in the hospital.

Analysis of the virulome of *S. marcescens* complex revealed a narrower range of features, with virulence genes related to flagella being the most common (Pérez-Viso et al., 2024). The intrinsic *cheY* gene, encoding proteins of chemotaxis, was present in most isolates, potentially delivering secondary signals to flagellar motors (Paul et al., 2010; Lazzaro, 2019). Furthermore, the *Yersinia* high-pathogenicity island, comprising genes related to the synthesis of the siderophore yersiniabactin (Carniel, 2001), was identified in *S. sarumanii* and *S. marcescens* in this study, and had previously been found in *S. liquefaciens* (Olsson et al., 2003). The transmission and exact virulence level of all these genes in *Serratia* spp. remain poorly understanded and require further investigation. Additionally, individual species contained unique genes compared with other three species, such as type II secretion system only presented in *S. sarumanii*.

In conclusion, we provided important insights into the population structure of *S. marcescens* complex were provided based on 106 isolates collected from ICUs in a single center over the past 11 years, representing five different species. Investigation into the potential transmissions of these *Serratia* isolates revealed that the majority of them were clonal related. Antimicrobial resistance analysis showed that while the overall incidence of resistance was not severe, carbapenemase-producing *S. marcescens* complex had emerged as early as 2014. To prevent the dissemination of these emergent and future clones, further research is needed to understand the transmission mechanisms and routes associated with these clinically significant *Serratia* clones.

## Data Availability

The original contributions presented in the study are publicly available. This data can be found here: BioProject accession number PRJNA1191627, with genome accession numbers JBMIOK000000000, JBMIOL000000000, and JBJNLA000000000 to JBJNOZ000000000.
