# Supplementary material for: Genomic diversity, antimicrobial resistance and dissemination of Serratia marcescens complex in patients admitted to ICUs
Source: Front Cell Infect Microbiol. 2025 Oct 16;15:1672468. doi: 10.3389/fcimb.2025.1672468 (PMC12571799; doi:10.3389/fcimb.2025.1672468)
Supplement: Supplementary file 1 [file Table1.docx]

**Supplementary materials**

**Genomic diversity, antimicrobial resistance and dissemination of *Serratia marcescens complex* in patients admitted to ICUs**

Table S1 Demographic and clinical characteristics of inpatient with *Serratia* spp. infections

|  | *S. bockelmannii* (n=8) | *S. marcescens* (n=12) | *S. sarumanii* (n=48) | *S. ureilytica* (n=16) |
| --- | --- | --- | --- | --- |
| Sex (%) |  |  |  |  |
| M | 62.5 | 66.7 | 70.8 | 43.8 |
| F | 37.5 | 33.3 | 29.2 | 56.3 |
| Age (year, IQR) | 53 (29-67) | 72 (64-83) | 68 (62-79) | 74 (70-82) |
| Department (%) |  |  |  |  |
| CICU | 25.0 | 16.7 | 2.1 | 12.5 |
| EICU | 25.0 | 16.7 | 16.7 | 25.0 |
| NICU | 25.0 | 25.0 | 33.3 | 37.5 |
| RICU | 12.5 | 16.7 | 27.1 | 18.8 |
| SICU | 12.5 | 41.7 | 20.8 | 6.3 |
| Median of Tmax (℃, IQR) | 38.4 (38.0-39.0) | 37.8 (37.2-38.6) | 38.0 (37.5-38.7) | 38.4 (37.9-39.1) |
| Fever (%) | 87.5 | 90.0 | 75.0 | 78.6 |
| Severe pneumonia (%) | 25.0 | 16.7 | 22.9 | 28.6 |
| Expectoration (%) | 100 | 66.7 | 75.0 | 92.9 |
| Cough (%) | 100 | 58.3 | 51.2 | 78.6 |
| Dyspnea (%) | 0 | 0 | 8.3 | 21.4 |
| Chill (%) | 0 | 0 | 6.3 | 14.3 |
| WBC count (10*^9^/L) | 75.0 | 75.0 | 66.7 | 60.0 |
| NGP (%) | 75.0 | 100 | 86.8 | 53.8 |
| Hemoglobin (g/L) | 75.0 | 75.0 | 80.6 | 76.9 |
| Death (%) | 0 | 16.7 | 0 | 18.8 |

ICU, intensive care unit; CICU, cardiac ICU; EICU, emergency ICU; NICU, neurology ICU; RICU, respiratory ICU; SICU, surgical ICU; WBC, white blood cell; NGP: neutrophilic granulocyte percentage.


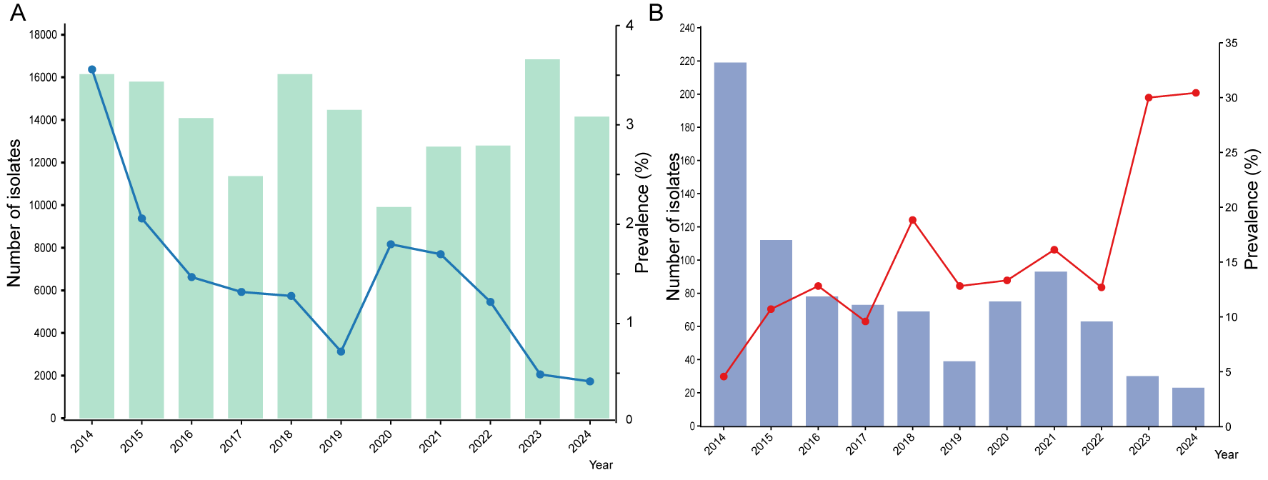


Figure S1 (A) The prevalence of *Serratia* spp. among the total bacterial isolates each year. The bar plots indicate the total number of clinical isolates each year. (B) The proportion *Serratia* spp. in ICUs compared to all *Serratia* clinical isolates from the entire hospital each year. The bar plots indicate the total number of *Serratia* spp. each year.


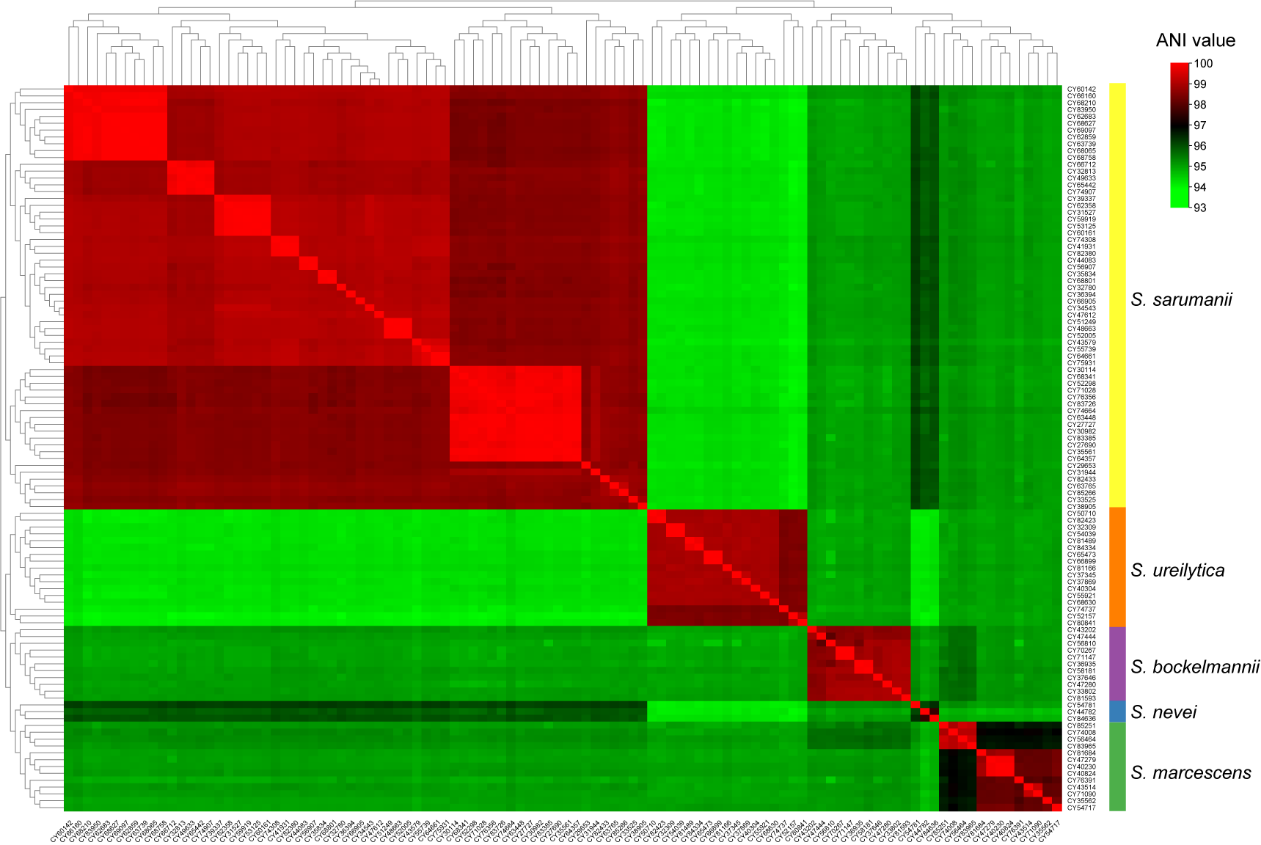


Figure S2 Heatmap of pairwise average nucleotide identity values for *Serratia* clinical isolates. Genomes similarities were obtained by calculating pairwise genetic distances using FastANI v1.34 (https://github.com/ParBLiSS/FastANI).


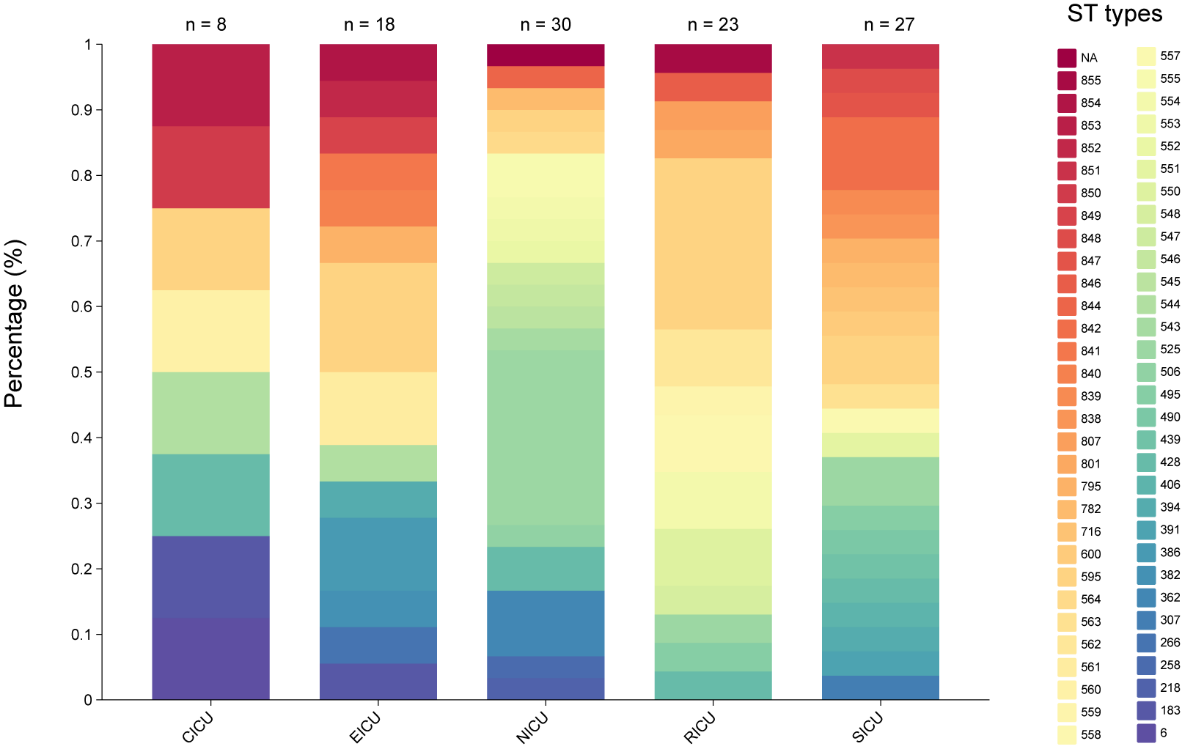


Figure S3 Proportions of each ST in each ICU. The colors of each ST are labeled on the right. The total number of isolates in each ICU shows in the top of each bar.


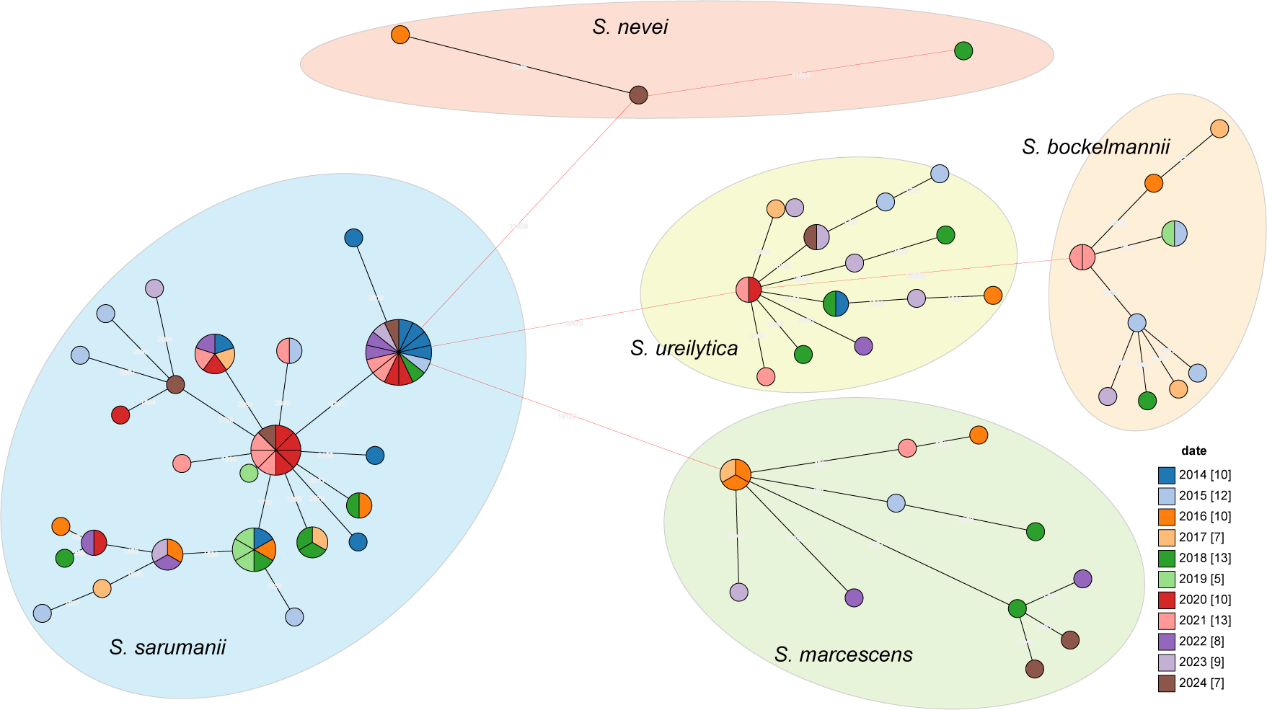


Figure S4 Minimum spanning trees based on core genome. The circles on the trees are color-coded to represent genetic clusters determined by less than 16 SNPs, with each color corresponding to the collection year. The numbers in brackets in the legend represent the number of strains.


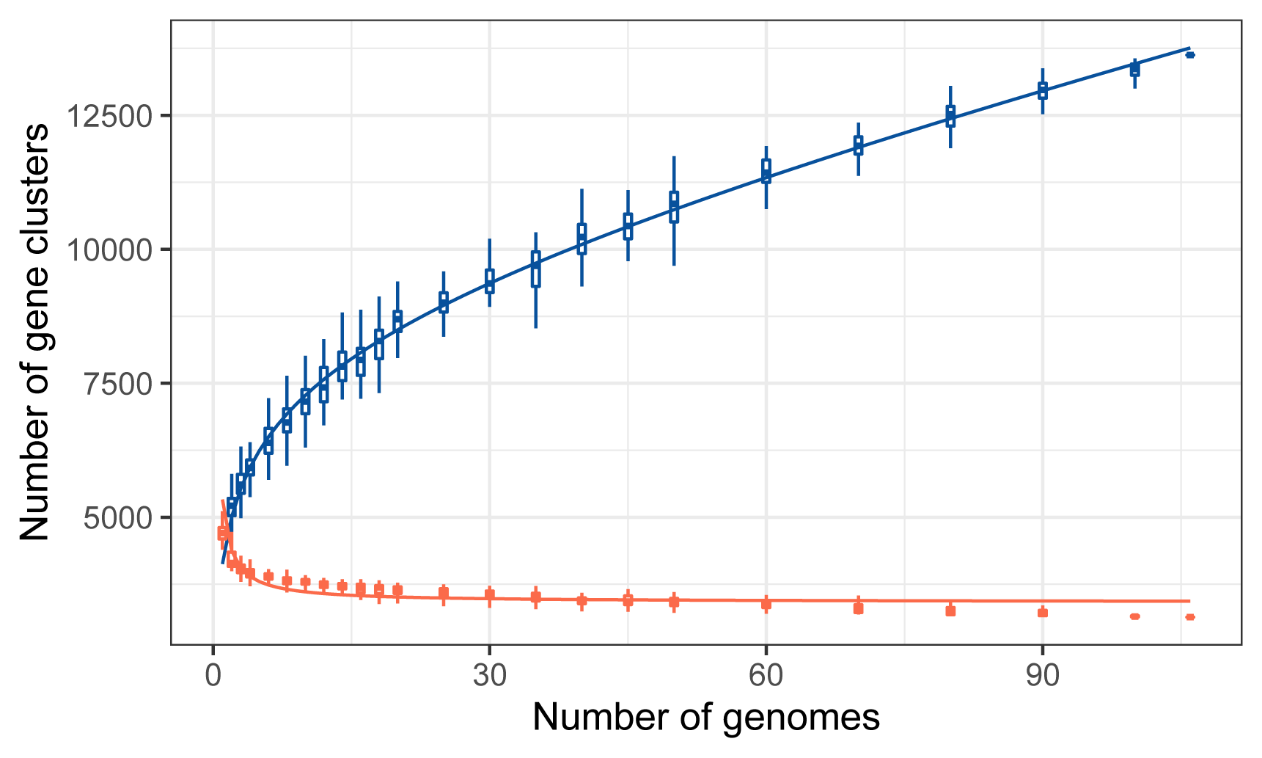


Figure S5 The rarefaction curve obtained from Roary analysis showing the distribution of core and accessory genes. The blue curve represents the number of accessory genes, while the orange curve shows the number of core genes.


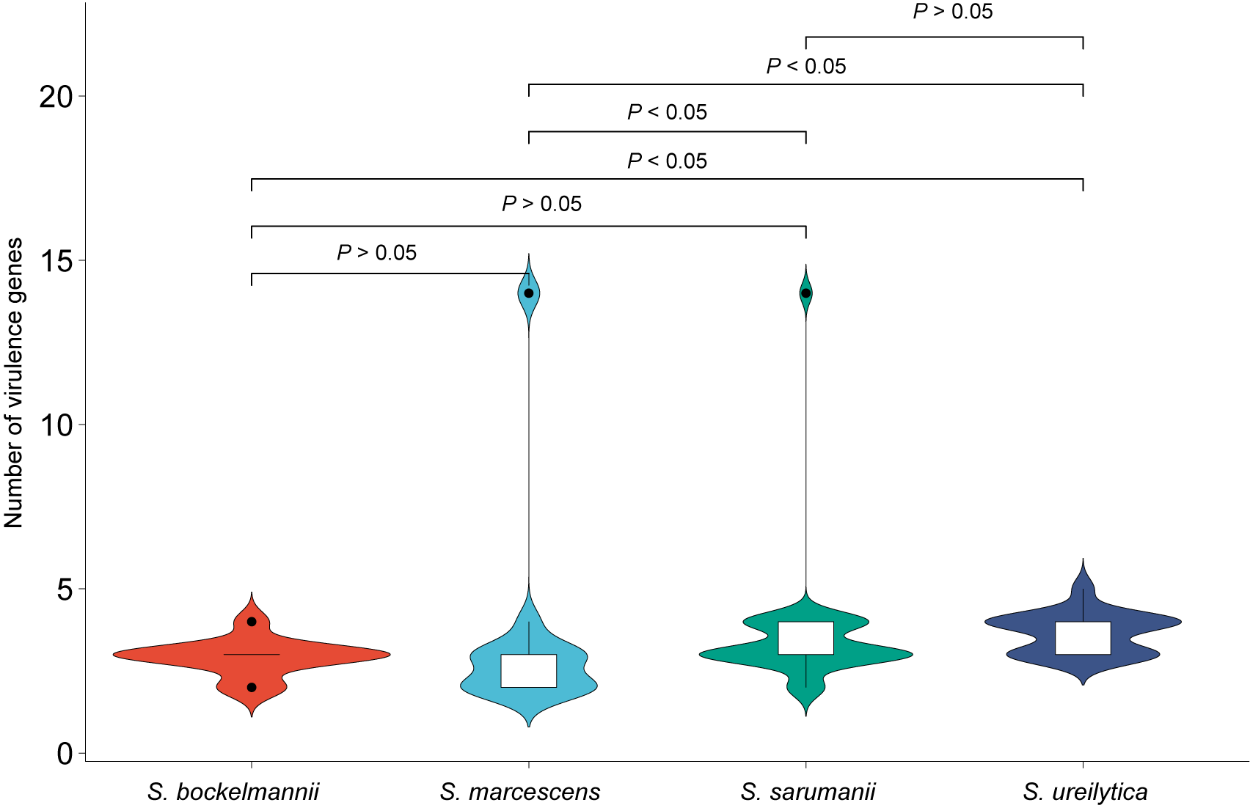


Figure S6 Violin plot displaying the number of virulence genes for each isolate within their respective species. The horizontal line within each box plot indicates the median number of virulence genes for each species. Significance values are labeled on the top of plot.


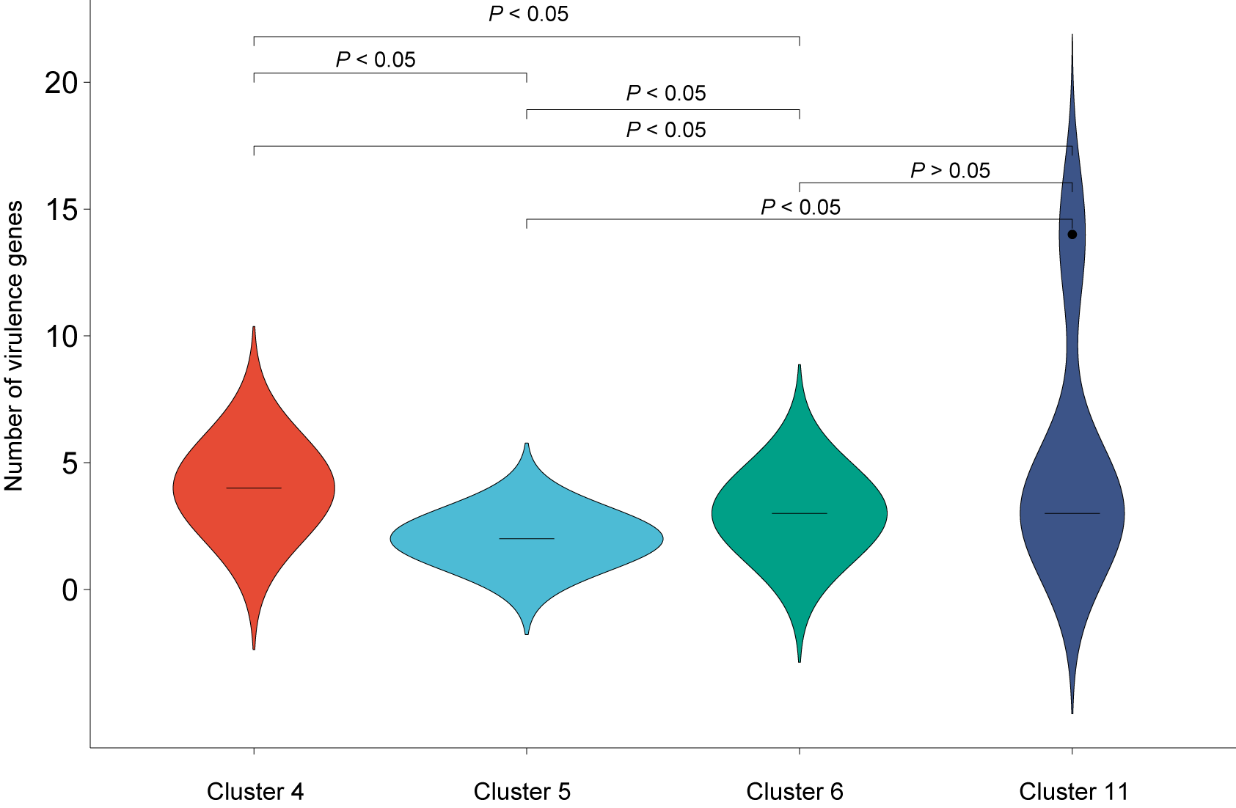


Figure S7 Violin plot revealing the number of virulence genes for each isolate within its corresponding cluster. The horizontal line within each box plot indicates the median number of virulence genes present in each cluster. Significance values are indicated at the top of the plot.
